# Expression of HNF4G and its potential functions in lung cancer

**DOI:** 10.18632/oncotarget.22933

**Published:** 2017-12-04

**Authors:** Jingyu Wang, Jun Zhang, Longsheng Xu, Ying Zheng, Danyan Ling, Zhiping Yang

**Affiliations:** ^1^ Department of Pathology, The First Affiliated Hospital of Jiaxing University, Jiaxing, China; ^2^ Department of Chest Surgery, The Second Affiliated Hospital of Jiaxing University, Jiaxing, China; ^3^ Department of Central laboratory, The First Affiliated Hospital of Jiaxing University, Jiaxing, China; ^4^ Department of Cell Division, Shanghai Emay Biotechnologies Co., Ltd, Shanghai, China; ^5^ Department of Oncology (04-F-14), The First Affiliated Hospital of Jiaxing University, Jiaxing, China

**Keywords:** HNF4G, AKT, lung cancer, proliferation, apoptosis

## Abstract

The hepatocyte nuclear factor 4 gamma (HNF4G), a member of orphan nuclear receptors, is up-regulated and functions as an oncoprotein in bladder cancer. In the present study, we observed that HNF4G expression was elevated in lung cancer tissues as compared to adjacent normal lung tissues. The expression of HNF4G protein was correlated with the tumor size and the prognosis of patients. Transfection with a small interference RNA (siRNA) targeting HNF4G in two lung cancer cell lines (H358 and H292 cells) significantly inhibited cell proliferation via arresting cells at G1 phase and inducing cell apoptosis. In addition, HNF4G siRNA reduced cell proliferation in a xenograft tumor-bearing model. Moreover, A549 cells, which had relative lower level of HNF4G, were ectopic expressed with HNF4G and treated with an AKT inhibitor (MK-2206). MK-2206 exposure not only attenuated the promoting effects of HNF4G overexpression on cell proliferation and cell cycle progression, but also suppressed the inhibitory effects of HNF4G overexpression on cell apoptosis. These data suggested that AKT signaling pathway was a potential upstream mediator of HNF4G. Collectively, our data indicate that HNF4G exerts as an oncogenic role in lung cancer by promoting cell proliferation and that HNF4G expression is a potential prognosis factor for lung cancer.

## INTRODUCTION

Lung cancer is one of the most lethal human malignancies with over 1.2 million death each year worldwide. The first risk factor for lung cancer is smoking [1]. Emerging evidence has demonstrated the alteration of components of several signaling pathways, such as epidermal growth factor receptor (EGFR) [2, 3], PI3K/AKT [4, 5], p53 [6, 7], p16^INK4^/cyclin D1/Rb pathways [8, 9], in lung cancer. Such alterations tend to drive cells to proliferation and escape from apoptosis. Therapy targeted cancer-related pathways has been an area of intense investigation and contributed to the improvement of cancer treatment [10–12].

The hepatocyte nuclear factor 4 (HNF4) belongs to the orphan nuclear receptor superfamily [13]. Two isoforms, HNF4A (NR2A1) and HNF4G (NR2A2), have been identified in mammals [14]. HNF4G was first identified in human kidney and HNF4G RNA is detected in the pancreas, kidney, small intestine and testis [15]. The physiological role of HNF4G has been studied by using *Hnf4g* knockout mice. Compared with littermate wild-type mice, *Hnf4g* knockout mice had decreased food intake, lower energy expenditure and locomotor activity [16], as well as improved glucose homeostasis [17]. Besides, the association between HNF4G and human diseases has also been investigated. Previous studies described that the mutations in HNF4G gene were not associated with the etiology of diabetes [18, 19]. A recent study reported that a polymorphism in HNF4G was associated with hyperuricemia in Chinese Han population [20]. Recently, Okegawa T et al. found that HNF4G expression is significantly elevated in bladder cancer and that HNF4G promotes the growth and invasion of bladder cancer partially by regulating HAS2 [21]. However, no studies have yet elucidated the expression and the role of HNF4A on lung carcinogenesis. It has been reported that AKT can regulate the transcriptional activity of several nuclear receptors, such as Nur77 [22], FOXO1 [23], FOXO3 [24] and HNF3B [25]. Whether AKT regulates HNF4G has far from understanding.

In the current study, we found that HNF4G expression was remarkably up-regulated in lung cancer tissues as compared with adjacent normal lung tissues. HNF4G expression level was associated with tumor size and overall survival rate. Genome Set Enrichment Analysis (GSEA) and biological function assays demonstrated that HNF4G might exert oncogenic role by promoting cell proliferation and cell cycle progression, as well as inhibiting cell apoptosis. Moreover, we proposed that AKT was involved in the regulation of HNF4G.

## RESULTS

### Expression of HNF4G in lung cancer tissues

**Figure 1:**
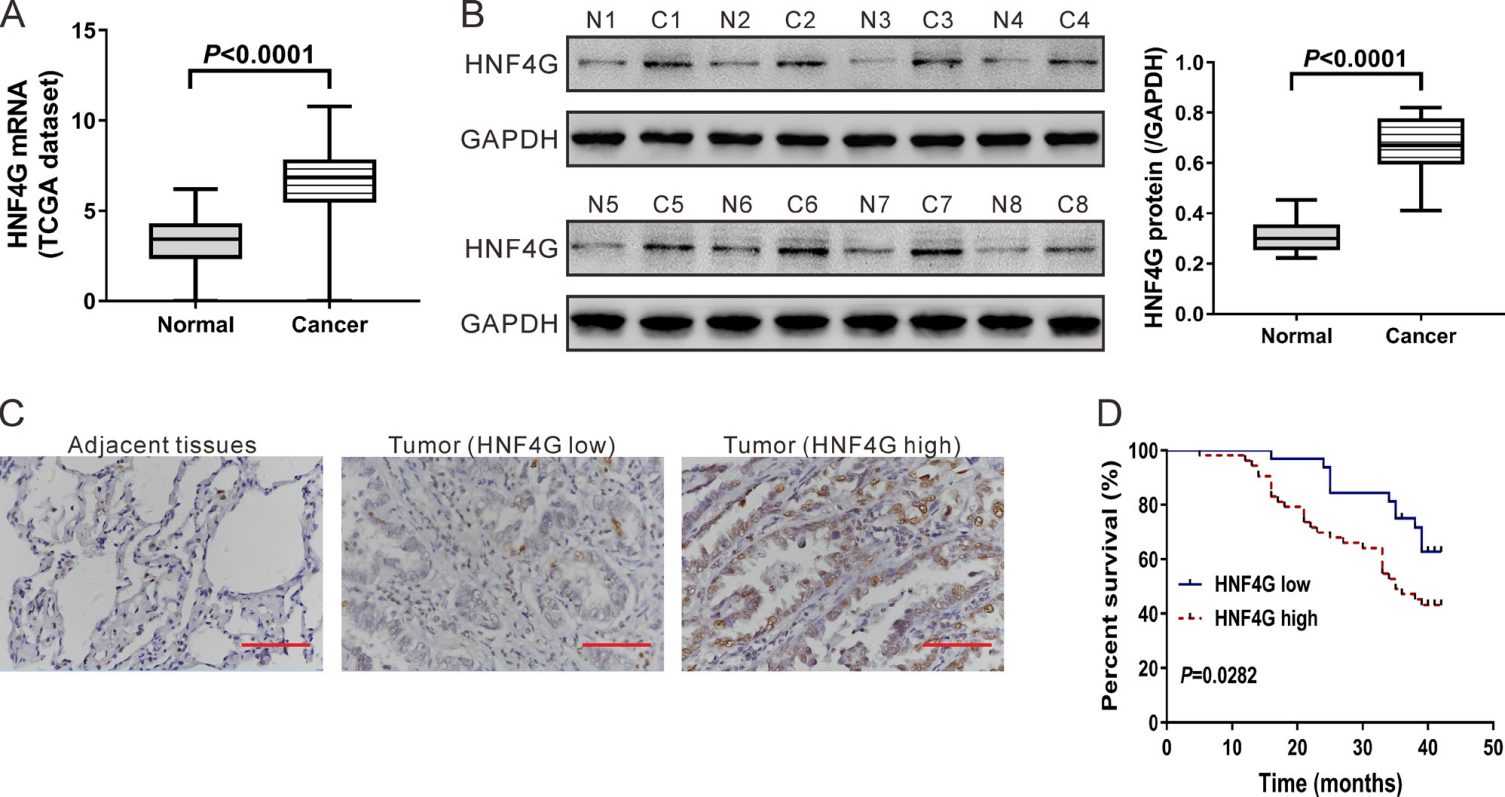
Expression of HNF4G in lung cancer tissues and normal lung tissues (**A**) HNF4G mRNA expression analysis based on TCGA dataset, which included 58 normal lung tissues and 488 lung cancer tissues. (**B**) Western blotting analysis of HNF4G and FOXO3 protein in tissue samples. Representative blot (left panel) and quantification of three independent experiments (right panel) were shown. T1-T8, tumor tissue, C1-C8, adjacent normal lung tissue. (**C**) Expression of HNF4G was determined by immunohistochemical staining in lung cancer and adjacent normal tissues (*n* = 85). Magnification: 400´. Scale bars: 50 μm. (**D**) Kaplan–Meier survival analyses of 85 patients with lung cancer. Survival analysis showed that HNF4G-low expression tumors (*n* = 32) had a favorable prognosis compared to HNF4G-high expression tumors (*n* = 53) (*P* < 0.01).

By analyzing the expression data of 488 lung cancer specimens and 58 normal lung specimens from The Cancer Genome Atlas project (TCGA, https://tcga-data.nci.nih.gov/tcga/), we found that HNF4G expression was significantly higher in lung cancer tissues (*P* < 0.0001; Figure 1A). To investigate HNF4G expression at translational level, we performed western blotting analysis on available 8 pairs of tissue samples and similar results were obtained (*P* < 0.0001; Figure 1B). To further verify this finding, we performed immunohistochemical staining on lung cancer tissues from 85 patients. More than 20% of tumor cells were positively stained in 53 cases, which were defined as HNF4G high expression group. In other 32 cases, less than 20% of tumor cells were positively stained, which were defined as HNF4G low expression group (Figure 1C).

### HNF4G expression and patient's overall survival

We next analyzed the relationship between HNF4G expression and clinicopathological characteristics of the 85 patients enrolled in this study. We found that the expression of HNF4G in lung cancer tissues was closely associated with tumor size (*P* < 0.05; Table 1). HNF4G expression had no significant association with other clinicopathological features, such as gender, age, tumor differentiation and lymph node metastases. Kaplan–Meier survival plots were generated based on HNF4G protein levels and follow-up records. Log-rank test revealed that the overall survival of patients with high HNF4G expression was worse than those with low HNF4G expression (*P* < 0.05; Figure 1D).

**Table 1:** Correlation of HNF4G expression with patients’ features

Variables		All cases	HNF4G protein		
			Low (*n* = 32)	High (*n* = 53)	*P* value
Gender	Male	44	18	26	0.6546
	Female	41	14	27	
Age (Years)	≥ 60	39	12	27	0.2623
	< 60	46	20	26	
Differentiation	Well	14	6	8	0.6455
	Moderate	48	16	32	
	Poor	23	10	13	
Tumor size (cm)	≥ 5	51	14	37	0.0175^*^
	< 5	34	18	16	
Lymph node metastasis	Positive	38	13	25	0.5565
	Negative	47	19	28	

^**^P < 0.01.

### HNF4G was strongly correlated with cell cycle and apoptosis pathways

**Figure 2:**
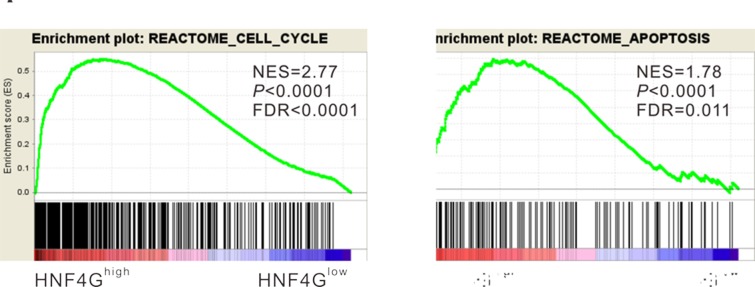
Enrichment plots of GSEA GSEA demonstrated the genes of Rectome cell cycle (**A**) and apoptosis (**B**) pathways were more correlated with patients with HNF4G^high^ versus HNF4G^low^ patients. NES, normalized enrichment score.

To identify pathways correlated with HNF4G, we performed Gene set enrichment analysis (GSEA) comparing lung cancer samples with high expression and low expression of HNF4G using TCGA dataset. The results showed that high expression of HNF4G was strongly correlated with the genes of Rectome cell cycle and apoptosis pathways (Figure 2). Deregulated cell cycle and apoptosis pathways play a key role during neoplastic progression [26]. Thus, we supposed that HNF4G may be involved in the progression of lung cancer.

### Knockdown of HNF4G inhibited the proliferation of lung cancer cells *in vitro* and *in vivo*

**Figure 3:**
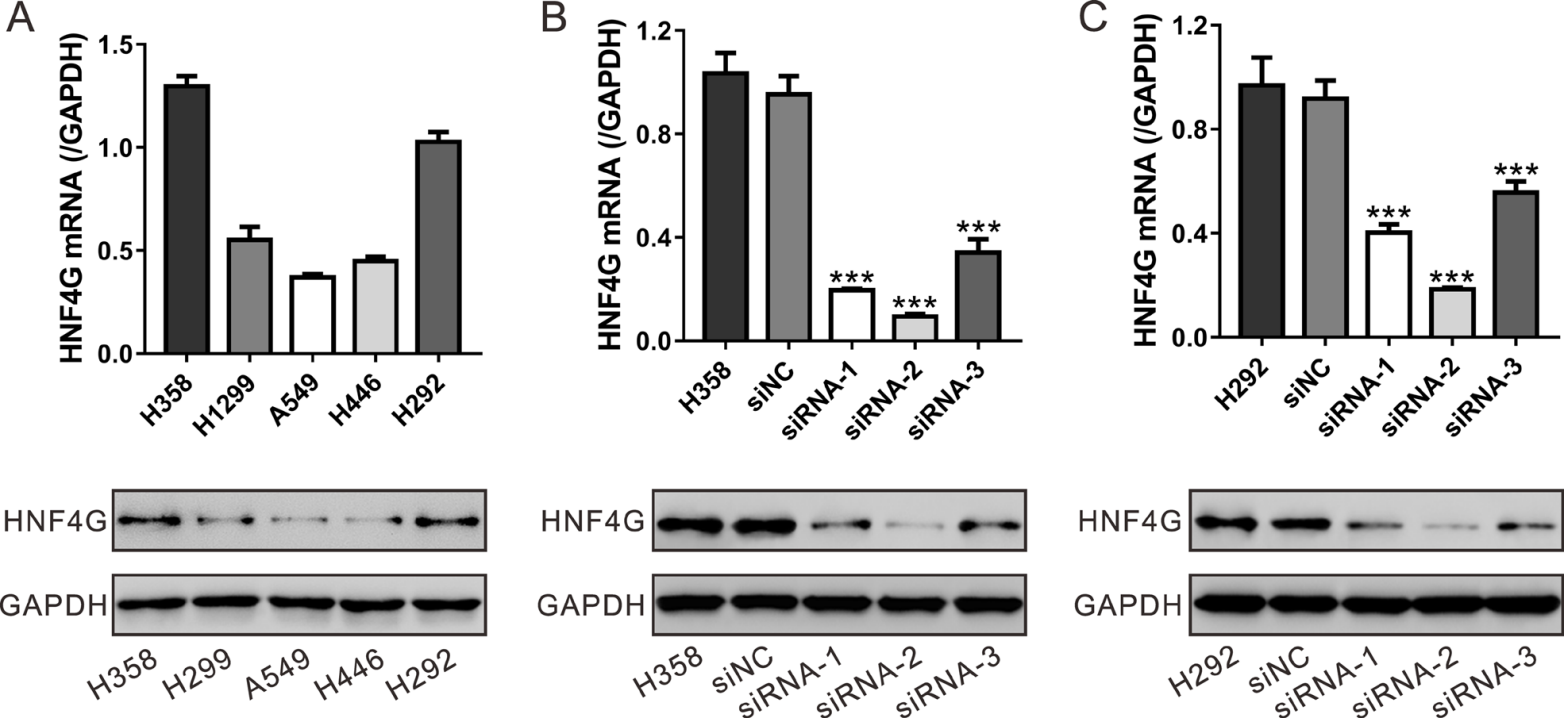
Suppressing of HNF4G expression by siRNA transfection (**A**) HNF4G expression level in five lung cancer cell lines was analyzed by qRT-PCR (upper panel) and western blot (lower panel). (**B**, **C**) The efficiency of three siRNAs in knocking down endogenous HNF4G in H358 (B) and H292 cells (C) was evaluated using qRT-PCR (upper panel) and western blot (lower panel). The experiments were repeated three times. ^***^P < 0.001 vs. siNC.

To investigate the biological functions of HNF4G in lung cancer, we transfected small interference RNAs (siRNAs) targeting HNF4G (siRNA-1, siRNA-2 and siRNA-3) or control siRNA (siNC) into H358 and H292 cells, which expressed relatively higher level of HNF4G (Figure 3A). As shown in Figure 3B and 3C, all three siRNAs caused a strong inhibitory effect on the mRNA and protein expression of HNF4G. siRNA-2, which showed higher knockdown efficiency than siRNA-1 and siRNA-3, was chosen for the following experiments.

**Figure 4:**
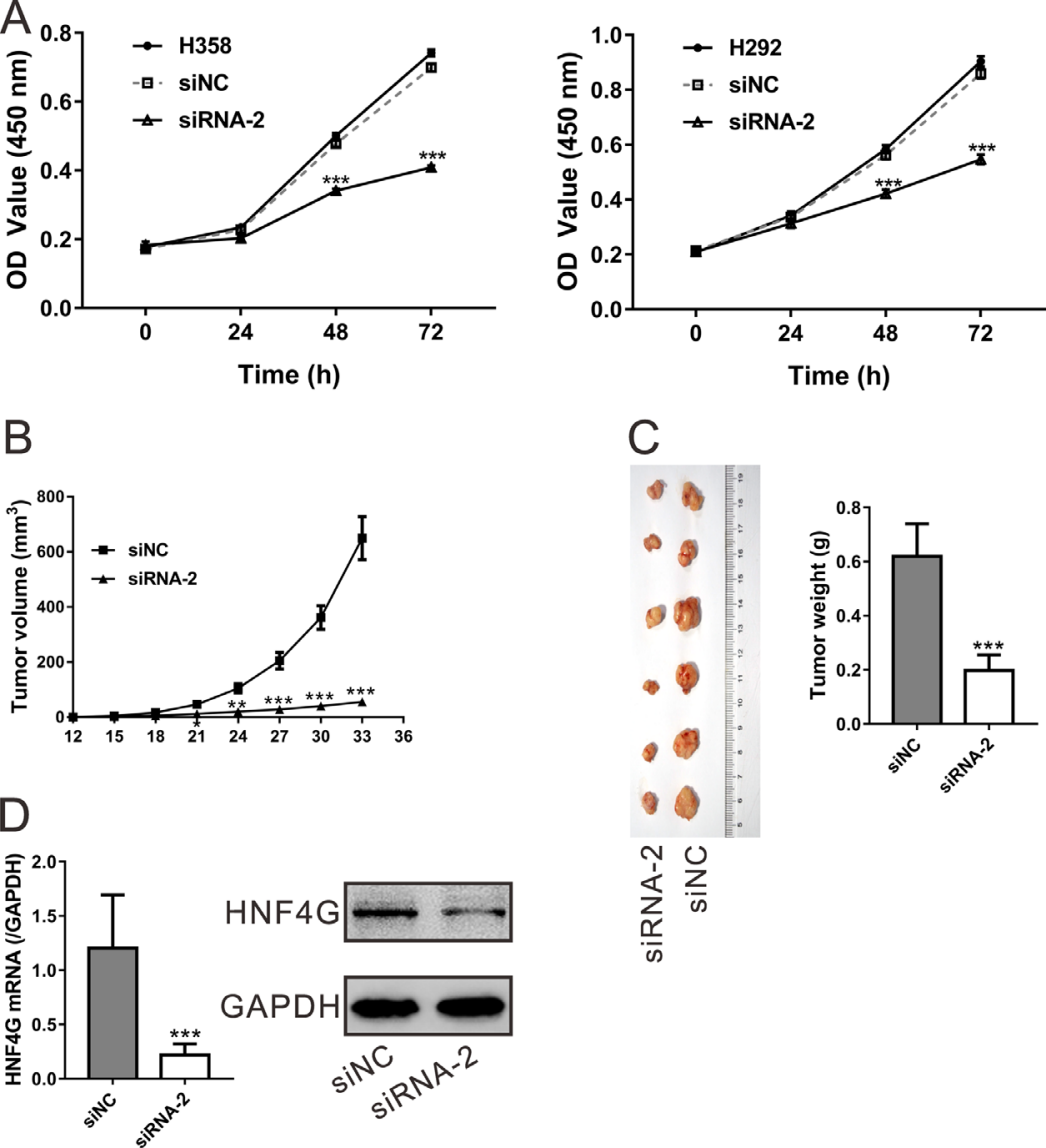
HNF4G knockdown suppressed the proliferation of lung cancer cells *in vitro* and *in vivo* (**A**) Cell proliferation was evaluated by CCK-8 assay when H358 and H292 cells were knocked down with HNF4G siRNA-2. The experiments were performed in triplicated and repeated three times. (**B**–**D**) HNF4G siRNA (siRNA-2) treatment significantly inhibited tumor growth in nude mice xenograft model as compared to siRNA control (siNC). After HNF4G siRNA-2 or siNC injection, the tumor volume (B) was measured every three days. At 33 days after cell inoculation, the tumors were resected and weighed. The pictures (left panel) and weight (right panel) of recovered tumors (*n* = 6) were shown (C). HNF4G (D) expression in xenograft tumors was determined by qRT-PCR (upper panel) and western blot (lower panel). The experiments were repeated three times and representative blots were presented. ^*^P < 0.05, ^**^P < 0.01, ^***^P < 0.001 vs. siNC.

Next, we conducted Cell Counting Kit-8 (CCK-8) assay to assess the effect of HNF4G knockdown on cell proliferation. The corresponding cell growth curves showed that optical density at 450 nm (OD450) of the cells transfected with siNC was not significantly different from those of cells without any treatment (Mock) (*P* > 0.05; Figure 4A). However, the OD450 values for the cells transfected with HNF4G siRNA-2 were significantly decreased at 48 and 72 h after transfection in both H358 and H292 cells as compared to siNC and Mock cells (*P* < 0.001).

We then explored whether HNF4G-siRNA-2 was able to inhibit tumor growth *in vivo*. Equal amount of H358 cells were subcutaneously injected into nude mice, and after tumor formation, the nude mice were intravenous injected with HNF4G siRNA-2 or siNC. As shown in Figure 4B, a slower tumor growth rate was observed in mice injected with HNF4G siRNA-2 as compared with mice injected with siNC. At 33 days after cell inoculation, the weight of HNF4G siRNA-2 treated tumors was less than 35% of that of siNC-treated tumors (Figure 4C). Meanwhile, tumors with HNF4G-siRNA-2 injection had a significant lower levels of HNF4G than those with siNC injection as indicated by quantitative real-time PCR (qRT-PCR) and western blot analyses (Figure 4D). These data suggested that inhibition of HNF4G in lung cancer cells repressed cell proliferation both *in vitro* and *in vivo*.

### Silencing of HNF4G arrested cells at G1 phase and induced cell apoptosis

**Figure 5:**
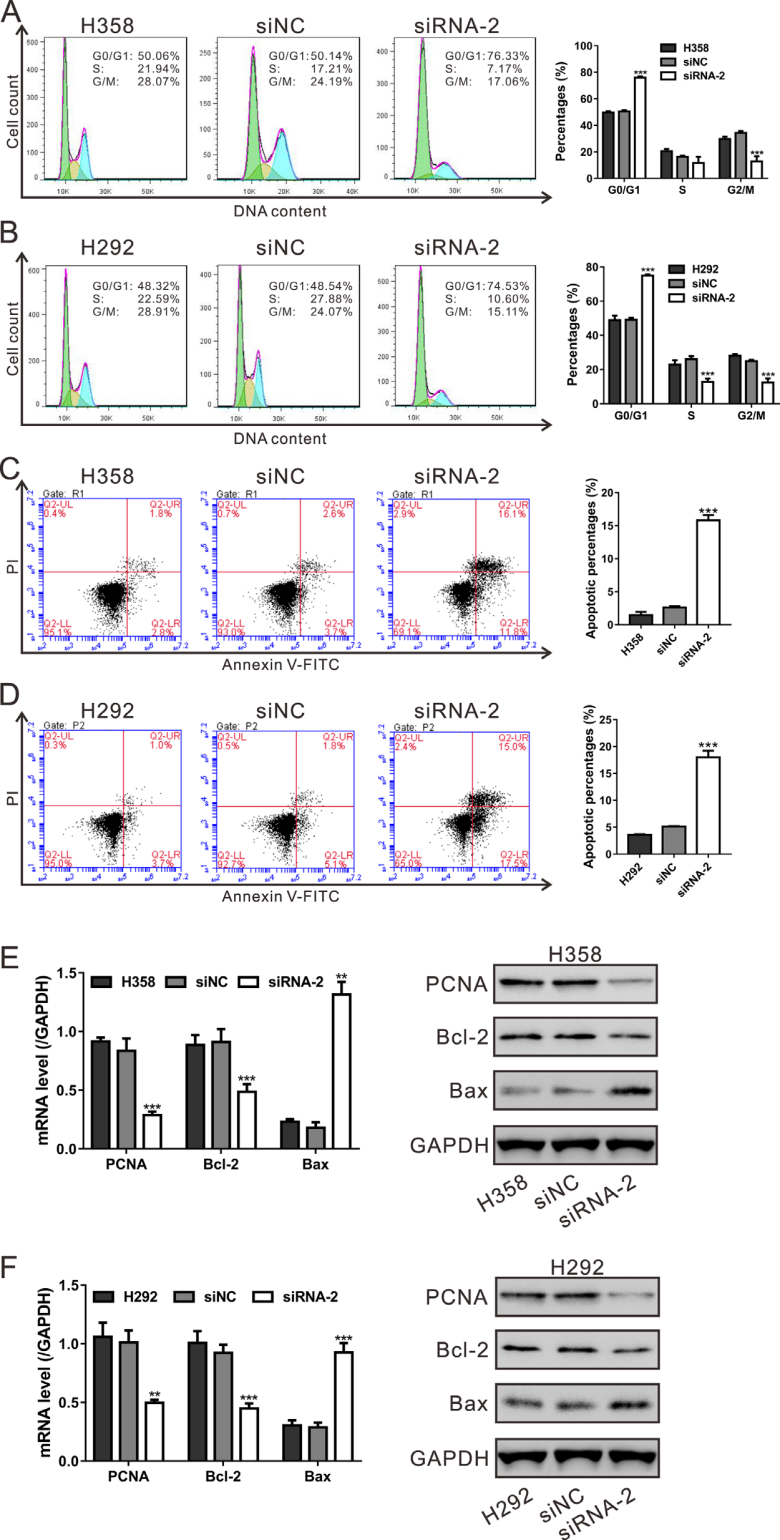
HNF4G knockdown induced cell cycle arrest and cell apoptosis of lung cancer cells (**A**, **B**) The synthesized siRNA-2 against HNF4G suppressed the G1-S phase transition in H358 (A) and H292 cells (B). Cell cycle distribution was determined by PI staining and flow cytometry analysis. The experiments were repeated three times and representative images were shown. (**C**, **D**) Apoptotic cells were measured by Annexin V-PI staining when H358 (C) and H292 cells (D) cells were knocked down with HNF4G siRNA-2. The experiments were repeated three times and representative images were shown. (**E**, **F**) mRNA and protein expression of cell cycle and apoptosis-related genes was determined by qRT-PCR (left panel) and western blot (right panel). ^**^P < 0.01, ^***^P < 0.001 vs. siNC.

The inhibited cell proliferation may be associated with the hampered cell cycle progression and the induced cell apoptosis. Considering the results of GSEA that HNF4G expression in lung cancer patients was closely associated with cell cycle and apoptosis pathways, we then evaluated the effects of HNF4G knockdown on cell cycle and apoptosis of H358 and H292 cells. As shown in Figure 5A and 5B, HNF4G siRNA-2 transfection led to a remarkable increase of cell percentages at G0/G1 phase, and a notable decrease of cell percentages at S-phase and/or G2/M phase (*P* < 0.001). As illustrated in Figure 5C and 5D, transfection with HNF4G siRNA-2 significantly increased the apoptotic ratio of cells compared to cells transfected with siNC (*P* < 0.001).

Furthermore, we applied qRT-PCR and western blotting assays to evaluate the mRNA and protein levels of PCNA, Bcl-2 and Bax. We found that the expression of PCNA and Bcl-2 was significantly decreased, and the expression of Bax was increased in both cell lines with HNF4G silenced (Figure 5E and 5F). These data indicated the effects of HNF4G on the transcription and translation of the cell cycle and cell apoptosis-related genes.

### Involvement of AKT pathway in the functions of HNF4G

**Figure 6:**
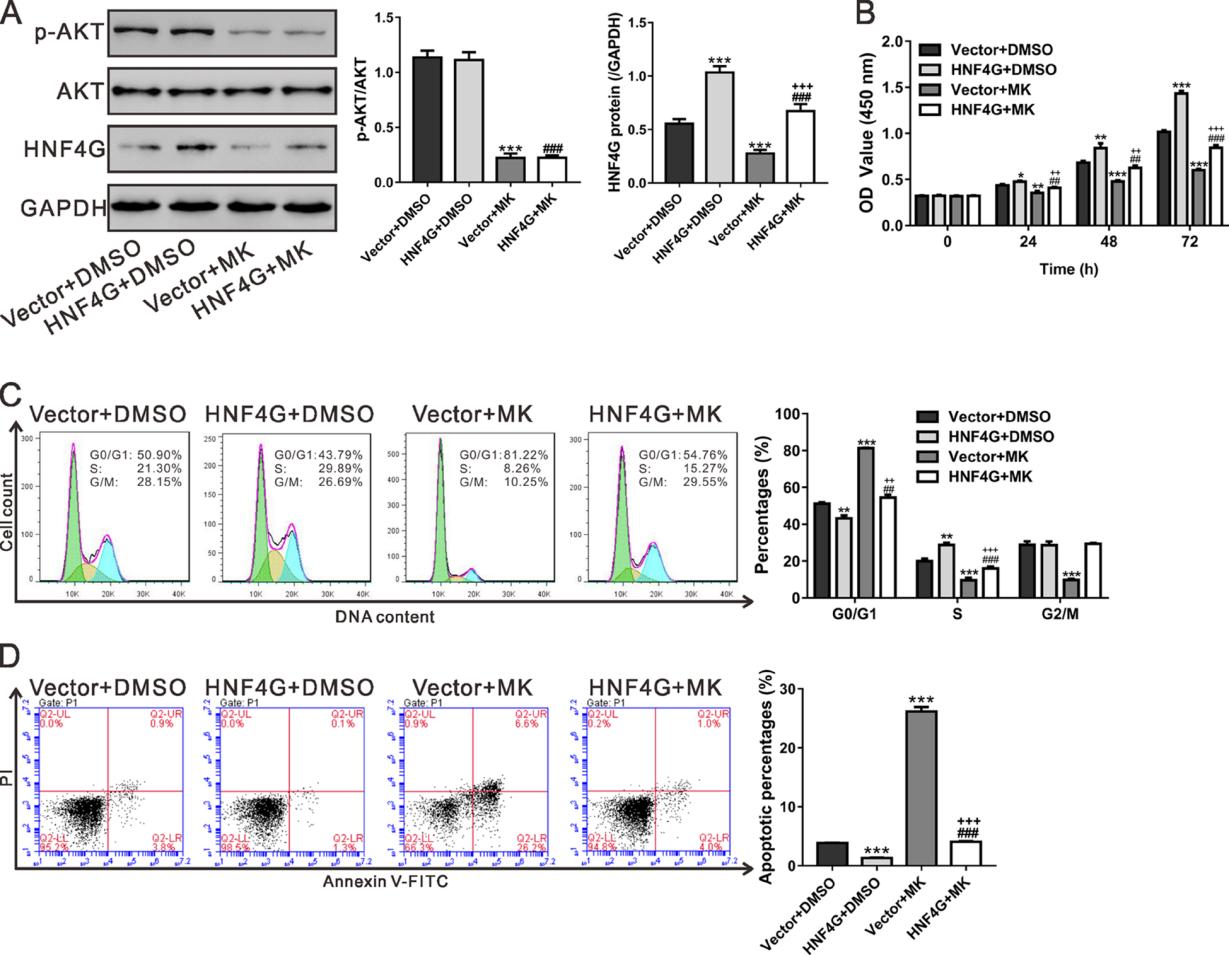
Involvement of AKT pathway in the functions of HNF4G A549 cells were infected with control virus (Vector) or HNF4G virus, and the protein levels of p-AKT, AKT and HNF4G (**A**), cell proliferation (**B**), cell cycle distribution (**C**) and cell apoptosis (**D**) were determined in the present of either DMSO or AKT inhibitor (MK-2206, 2 μM). The experiments were repeated three times and representative images were shown. ^*^P < 0.05, ^**^P < 0.01, ^***^P < 0.001 versus Vector^+^DMSO; ^##^*P* < 0.01, ^###^*P* < 0.001 versus HNF4G^+^DMSO; ^++^*P* < 0.01, ^+++^*P* < 0.001 versus Vector^+^MK-2206.

AKT/PKB, a key signaling transduction protein related with cell survival and apoptosis, is frequently overactivated in tumor cells [27]. To clarify the contribution of AKT signaling, A549 cells, which displayed a relative low level of HNF4G (Figure 2A), were infected with HNF4G overexpression virus and treated with an AKT inhibitor (MK-2206). HNF4G overexpression virus significantly enhanced HNF4G expression, while MK-2206 showed an inhibitory effect in HNF4G expression (Figure 6A and Supplementary Figure 1). HNF4G overexpression induced cell proliferation (Figure 6B) and cell cycle progression (Figure 6C), but inhibited cell apoptosis (Figure 6D). MK-2206 treatment had reversed effects. Additionally, MK-2206 exposure attenuated the effects of HNF4G overexpression on A549 cells. These data showed that AKT pathway was a potential upstream regulator of HNF4G in lung cancer.

## DISCUSSION

In this study, we investigated the expression and biological functions of a nuclear receptor, HNF4G in lung cancer. First, we studied the expression of HNF4G in lung cancer tissues. HNF4G mRNA and protein expression was frequently up-regulated in lung cancer tissues compared to normal lung tissues (Figure 1). We then analyzed the relation between HNF4G protein expression and clinicopathological features, and found that higher expression of HNF4G was correlated with larger tumor size and poorer overall survival (Table 1 and Figure 1D). Although further study with large sample size is needed to clarify the relation, our study suggests the potential clinical value of HNF4G in lung cancer.

Next, we investigated the biological functions of HNF4G in lung cancer. Functional assays showed that RNA interference-mediated inhibition of HNF4G significantly repressed lung cancer cell proliferation *in vitro* and *in vivo* (Figure 4). On the contrary, HNF4G overexpression stimulated lung cancer cell proliferation (Figure 6B). These findings indicate that up-regulated expression of HNF4G may promote cell growth and tumorigenicity of lung cancer, which was in line with the previous study on bladder cancer [21]. By GSEA analysis on TCGA dataset, we identified that HNF4G expression was positively correlated with genes in cell cycle and apoptosis pathways (Figure 2), which was confirmed by *in vitro* experiments with HNF4G silence or overexpression (Figure 5 and Figure 6). Additionally, down-regulation of HNF4G led to a reduction in the protein and mRNA levels of PCNA (a proliferation marker [28]) and Bcl-2 (an anti-apoptosis factor [29]), and an augment in those of Bax (a proapoptotic factor [29]) (Figure 5). Our data indicate that HNF4G exerts proliferation-promoting effect on lung cancer cells via speeding up cell cycle transition and inhibiting cell apoptosis.

AKT, a key signaling transduction protein related with cell survival and apoptosis, has been reported overactive in lung cancer [4, 5]. AKT has been reported to phosphorylate and regulate the transcriptional activity of several nuclear receptors, such as Nur77 [22], FOXO1 [23], FOXO3 [24] and HNF3B [25]. In the present study, AKT inhibitor (MK-2206) inhibited the transcription of HNF4G. Additionally, MK-2206 exposure attenuated the proliferation-promoting and apoptosis-inhibitory effects of HNF4G overexpression on A549 cells (Figure 6). Our results suggests that AKT pathway is a potential upstream regulator of HNF4G in lung cancer. Further investigation is required to clarify whether HNF4G is regulated by AKT directly or indirectly, and to bring insight into the mechanisms how AKT is involved in the functions of HNF4G.

Collectively, our study suggests that HNF4G is up-regulated in human lung cancer specimens and may be a prognostic marker for lung cancer. HNF4G functions actively in the promotion of cell proliferation and cell cycle progression, the inhibition of cell apoptosis in lung cancer, and AKT signal pathway is possible to be involved in the biological function of HNF4G.

## MATERIALS AND METHODS

### Patients and sample collection

The study protocol was approved by Research Ethic Committee in The First Affiliated Hospital of Jiaxing University. A total of 85 patients who underwent surgical resection of lung cancer at The First Affiliated Hospital of Jiaxing University between January 2010 and December 2013 were enrolled in this study. Written informed consent was obtained from all enrolled patients. Patients who had received preoperative chemotherapy or radiotherapy were excluded from the study. The enrolled patients included 44 males and 41 females, with an average age of 59. The clinical and pathological information, and survival data were obtained by review of medical records and follow-up information. Archived paraffin-embedded tissue samples of lung cancer from all 85 patients and adjacent normal lung tissue samples were processed for immunohistochemical staining of HNF4G. Eight pairs of fresh samples including tumor tissues and the adjacent normal tissues were used for detection of HNF4G protein levels by western blotting.

### Immunohistochemical staining

The paraffin-embedded tissue samples were cut into 5 μm-thick sections. The sections were deparaffinized, rehydration, and then antigen-retrieved by heating in 0.01 M citrate buffer (pH 6.0) for 15 min. Following treating with 0.3% hydrogen peroxide for 15 min, the sections were incubated with 10% normal goat serum for 30 min and then with HNF4G antibody (Abcam, Cambridge, MA, USA) overnight at 4°C. Then, horseradish peroxidase (HRP) conjugated secondary antibody was applied for 1 h at room temperature. The sections were stained with the 3,3-diaminobenzidine solution (Long island, Shanghai, China) and counterstained with hematoxylin (BASO, Zhuhai, China). The patients were graded into HNF4G low expression group (positive stain was observed in less than 20% of tumor cells) and HNF4G high expression group (positive stain was observed in more than 20% of tumor cells).

### Western blotting

Total proteins were extracted using Radio-immunoprecipitation assay (RIPA) lysis buffer containing phenylmethanesulfonyl fluoride (PMSF; Solarbio, Beijing, China). The protein concentrations were determined by BCA protein assay kit (Thermo Fisher Scientific). Equal amount of protein (30 μg) were resolved by 10% sodium dodecyl sulfate polyacrylamide gel electrophoresis (SDS-PAGE). After transferring onto nitrocellulose membranes (Millipore, Bredford, MA, USA), the blots were blocked in 5% skim milk at room temperature for 1 h, and then probed with diluted primary antibodies overnight at 4°C. After washing three times with TBST, the blots were incubated with HRP-labelled secondary antibodies (Beyotime, Shanghai, China) at room temperature for 1 h. Following three washes with TBST, the membranes were developed using a chemiluminescence detection kit (Millipore). GAPDH was used as an endogenous reference. The resources of primary antibodies were as follows: Abcam (Cambridge, MA, USA), anti-HNF4G; Cell Signaling Technology (Danvers, MA, USA), anti-PCNA, anti-p-AKT, anti-AKT and anti-GAPDH; Santa Cruz Biotech. (Santa Cruz, CA, USA), anti-Bcl-2 and anti-Bax.

### RNA extraction and quantitative real-time PCR (qRT-PCR)

Total RNA was isolated using TRIzol reagent (Invitrogen, Carlsbad, CA, USA) per the manufacturer's recommendations. After treated with DNase I, total RNA was reverse transcribed to complementary DNA (cDNA) with the Reverse Transcription kit (Fermentas, Hanover, MD, USA). qRT-PCR was conducted with SYBR Grenn qPCR Master Mix (Thermo Fisher Scientific, Rockford, IL, USA) in conjunction with an ABI 7300 thermal cycler (Applied Biosystem, Foster City, CA, USA), according to the manufacturer's instructions. The sequences of primer sets were listed in Table 2. qRT-PCRs were performed in triplicates and mean cycle threshold (Ct) values were calculated for the expression analysis. GAPDH was served as an internal control for normalization. The mRNA expression levels of target genes were calculated by 2^−ΔΔCt^ method.

**Table 2:** Primers for the detection of mRNA expression

Primer	Primer sequence	Size (bp)
HNF4G (NM_001330561)	F: 5′- ACAGAATAAGCACCAGAAG −3′ R: 5′- TCACAGACATCACCAATAC −3′	163
PCNA (NM_002592)	F: 5′- CCTGCTGGGATATTAGCTCCA −3′ R: 5′- CAGCGGTAGGTGTCGAAGC −3′	109
Bcl-2 (NM_000633)	F: 5′- GGTGGGGTCATGTGTGTGG −3′ R: 5′- CGGTTCAGGTACTCAGTCATCC −3′	89
Bax (NM_001291428)	F: 5′- CCCGAGAGGTCTTTTTCCGAG −3′ R: 5′- CCAGCCCATGATGGTTCTGAT −3′	155
GAPDH (NM_001256799)	F: 5′- CACCCACTCCTCCACCTTTG −3′ R: 5′- CCACCACCCTGTTGCTGTAG −3′	110

### GSEA

Publicly available dataset was downloaded from The Cancer Genome Atlas project (TCGA, https://tcga-data.nci.nih.gov/tcga/). GSEA was conducted using the GSEA desktop application (http://www.broadinstitute.org/gsea/index.jsp) as previously described [30].

### Cell culture

Human lung cancer cell lines H358, H1299, A549, H446 and H292 were obtained from the Cell Bank of the Chinese Academy of Sciences (Shanghai, China). All cell lines were maintained in RPMI-1640 medium (Hyclone, Logan, UT, USA) containing antibiotics and 10% fetal bovine serum (FBS; GIBCO, Carlsbad, CA, USA) at 37°C in a 5% CO2 humidified atmosphere.

### siRNA transfection

Three HNF4G siRNAs (siRNA-1: 5′- GGCCAAUCGUGUUCUAGAU −3′, siRNA-2: 5′- GGCUAAGCGAUCCAGUAAA −3′ and siRNA-3: 5′- GACCCAUUAACUGGACAAA-3′) and a control siRNA (siNC) were designed and synthesized by Genepharma (Shanghai, China). H358 and H292 cells were transiently transfected with the indicated siRNA using Lipofectamine 2000 (Invitrogen, Carlsbad, CA, USA) in accordance with the manufacture's protocol. Knockdown efficiency were checked by analyzing the mRNA and protein levels of HNF4G at 48 h after transfection.

### HNF4G overexpression lentivirus

The complete coding sequence of human HNF4G was cloned into the EcoRI and BamHI sites of the expression vector pLVX-puro (Clontech, Palo Alto, CA, USA) and confirmed by sequencing. HNF4G expressing lentivirus (HNF4G) and control lentivirus (Vector) were generated by co-transfection into HEK293 cells with helper plasmids.

### CCK-8 assay

The cell proliferation assay was conducted with the CCK-8 solution (SAB biotech. College Park, MD, USA) following the manufacturer's protocol. Briefly, H358, H292 and A549 cells were seeded to 6-well plates (5 × 10^5^ cells/well) and cultured to 60–70% confluence. H358 and H292 cells were transfected with siRNA-2/siNC, while A549 cells were transduced with overexpression/vector virus. At 6 h after transfection, cells were digested with trypsin and seeded in triplicate into 96-well plates (2 × 10^3^ cells/well). A549 cells were treated with DMSO/2mM MK-2206 (Aladdin, Shanghai, China). Following treatment, each well was incubated with 10 μl/well of Cell Counting Kit-8 solution for 2 h daily for 4 consecutive days. Optical density at 450 nm was measured on a microplate reader.

### Cell cycle and apoptosis assays

H358, H292 and A549 cells were seeded to 6-well plates (5 × 10^5^ cells/well) and cultured to 60–70% confluence. H358 and H292 cells were transfected with siRNA-2/siNC, while A549 cells were transduced with overexpression/vector virus and treated with DMSO/2 mM MK-2206. At 48 h after treatment, lung cancer cells were harvested, washed with ice-cold PBS and stained according to the manufacturers’ instructions. For cell cycle distribution analysis, the cells were fixed in cold ethanol overnight, rehydrated and stained with propidium iodide (PI)-RNase A solution (Sigma) in the dark at 37°C for 30 min. For apoptosis analysis, the cells were labeled with Annexin V-fluorescein isothiocyanate (FITC) and PI (Beyotime) in the dark at 4°C for 20 min. DNA content and cell apoptosis was then analyzed by a flow cytometer (BD Biosciences, Franklin Lakes, NJ, USA).

### Xenograft tumor-bearing model

The animal experiments were performed according to protocols approved by the Animal Care and Use Committee of Jiaxing University. Healthy BALB/c nude mice (4–5 weeks, *n* = 12) purchased from SLAC Animal (Shanghai, China) were maintained in specific pathogen-free status and used after 1 week of acclimatization. Xenograft tumor-bearing model was established by subcutaneously injection of H358 cells (4 × 10^6^ cells/mouse). Ten days after cell inoculation, the mice were randomly divided into siRNA-2 group and siNC group (*n* = 6/group). The mice were intravenous injected with siRNA-2 and siNC containing formulations, respectively, twice a week. Tumor volume (mm^3^) was calculated every three days by the following standard formula: a^2^ × b × 0.5, where a is the shortest diameter and b is the longest diameter of the tumor. Thirty-three days after cell inoculation, the mice were killed under anesthesia, and the tumors were resected and weighed. The mRNA and protein expression of HNF4G in tumors was examined.

### Statistical analysis

Statistical testing was conducted with Graphpad Prism software (version 6.0, San Diego, CA, USA). Kaplan-Meier survival curves were drawn and the log-rank test was applied to determine the differences between the groups. Student's t test and one-way analysis of variance (ANOVA) followed by Tukey's test were used to analyze data between two groups and between more than two groups, respectively. Statistical significance was set at *P* < 0.05.

## SUPPLEMENTARY MATERIALS FIGURES



## References

[1] Siegel RL, Miller KD, Jemal A (2015). Cancer statistics, 2015. CA Cancer J Clin.

[2] Hirsch FR, Varella-Garcia M, Bunn PA, Di Maria MV, Veve R, Bremmes RM, Baron AE, Zeng C, Franklin WA (2003). Epidermal growth factor receptor in non-small-cell lung carcinomas: correlation between gene copy number and protein expression and impact on prognosis. J Clin Oncol.

[3] Nicholson R, Gee J, Harper M (2001). EGFR and cancer prognosis. Eur J Cancer.

[4] Brognard J, Clark AS, Ni Y, Dennis PA (2001). Akt/protein kinase B is constitutively active in non-small cell lung cancer cells and promotes cellular survival and resistance to chemotherapy and radiation. Cancer Res.

[5] Tang JM, He QY, Guo RX, Chang XJ (2006). Phosphorylated Akt overexpression and loss of PTEN expression in non-small cell lung cancer confers poor prognosis. Lung Cancer.

[6] Olivier M, Petitjean A, Marcel V, Petre A, Mounawar M, Plymoth A, De Fromentel C, Hainaut P (2009). Recent advances in p53 research: an interdisciplinary perspective. Cancer Gene Ther.

[7] Brambilla E, Gazzeri S, Lantuejoul S, Coll JL, Moro D, Negoescu A, Brambilla C (1998). p53 mutant immunophenotype and deregulation of p53 transcription pathway (Bcl2, Bax, and Waf1) in precursor bronchial lesions of lung cancer. Clin Cancer Res.

[8] Brambilla E, Moro D, Gazzeri S, Brambilla C (1999). Alterations of expression of Rb, p16INK4A and cyclin D1 in non-small cell lung carcinoma and their clinical significance. J Pathol.

[9] Ding L, Getz G, Wheeler DA, Mardis ER, McLellan MD, Cibulskis K, Sougnez C, Greulich H, Muzny DM, Morgan MB (2008). Somatic mutations affect key pathways in lung adenocarcinoma. Nature.

[10] Antonicelli A, Cafarotti S, Indini A, Galli A, Russo A, Cesario A, Lococo FM, Russo P, Mainini AF, Bonifati LG (2013). EGFR-targeted therapy for non-small cell lung cancer: focus on EGFR oncogenic mutation. Int J Med Sci.

[11] Sawyers C (2004). Targeted cancer therapy. Nature.

[12] Huang M, Shen A, Ding J, Geng M (2014). Molecularly targeted cancer therapy: some lessons from the past decade. Trends Pharmacol Sci.

[13] Bertrand S, Brunet FG, Escriva H, Parmentier G, Laudet V, Robinson-Rechavi M (2004). Evolutionary genomics of nuclear receptors: from twenty-five ancestral genes to derived endocrine systems. Mol Biol Evol.

[14] Maglich JM, Sluder A, Guan X, Shi Y, McKee DD, Carrick K, Kamdar K, Willson TM, Moore JT (2001). Comparison of complete nuclear receptor sets from the human, Caenorhabditis elegans and Drosophila genomes. Genome Biol.

[15] Drewes T, Senkel S, Holewa B, Ryffel GU (1996). Human hepatocyte nuclear factor 4 isoforms are encoded by distinct and differentially expressed genes. Mol Cell Biol.

[16] Gerdin AK, Surve VV, Jonsson M, Bjursell M, Bjorkman M, Edenro A, Schuelke M, Saad A, Bjurstrom S, Lundgren EJ, Snaith M, Fransson-Steen R, Tornell J (2006). Phenotypic screening of hepatocyte nuclear factor (HNF) 4-gamma receptor knockout mice. Biochem Biophys Res Commun.

[17] Baraille F, Ayari S, Carrière V, Osinski C, Garbin K, Blondeau B, Guillemain G, Serradas P, Rousset M, Lacasa M (2015). Glucose Tolerance Is Improved in Mice Invalidated for the Nuclear Receptor HNF-4γ: A Critical Role for Enteroendocrine Cell Lineage. Diabetes.

[18] Hara M, Wang X, Paz VP, Cox NJ, Iwasaki N, Ogata M, Iwamoto Y, Bell GI (2000). No diabetes-associated mutations in the coding region of the hepatocyte nuclear factor-4gamma gene (HNF4G) in Japanese patients with MODY. Diabetologia.

[19] Plengvidhya N, Antonellis A, Wogan LT, Poleev A, Borgschulze M, Warram JH, Ryffel GU, Krolewski AS, Doria A (1999). Hepatocyte nuclear factor-4gamma: cDNA sequence, gene organization, and mutation screening in early-onset autosomal-dominant type 2 diabetes. Diabetes.

[20] Chen BD, Chen XC, Pan S, Yang YN, He CH, Liu F, Ma X, Gai MT, Ma YT (2017). TT genotype of rs2941484 in the human HNF4G gene is associated with hyperuricemia in Chinese Han men. Oncotarget.

[21] Okegawa T, Ushio K, Imai M, Morimoto M, Hara T (2013). Orphan nuclear receptor HNF4G promotes bladder cancer growth and invasion through the regulation of the hyaluronan synthase 2 gene. Oncogenesis.

[22] Pekarsky Y, Hallas C, Palamarchuk A, Koval A, Bullrich F, Hirata Y, Bichi R, Letofsky J, Croce CM (2001). Akt phosphorylates and regulates the orphan nuclear receptor Nur77.

[23] Tang ED, Nunez G, Barr FG, Guan KL (1999). Negative regulation of the forkhead transcription factor FKHR by Akt. J Biol Chem.

[24] Brunet A, Bonni A, Zigmond MJ, Lin MZ, Juo P, Hu LS, Anderson MJ, Arden KC, Blenis J, Greenberg ME (1999). Akt promotes cell survival by phosphorylating and inhibiting a Forkhead transcription factor. Cell.

[25] Wolfrum C, Besser D, Luca E, Stoffel M (2003). Insulin regulates the activity of forkhead transcription factor Hnf-3beta/Foxa-2 by Akt-mediated phosphorylation and nuclear/cytosolic localization. Proc Natl Acad Sci USA.

[26] Evan GI, Vousden KH (2001). Proliferation, cell cycle and apoptosis in cancer. Nature.

[27] Vivanco I, Sawyers CL (2002). The phosphatidylinositol 3-Kinase AKT pathway in human cancer. Nat Rev Cancer.

[28] Hall P, Levison D, Woods A, Yu CW, Kellock D, Watkins J, Barnes D, Gillett C, Camplejohn R, Dover R (1990). Proliferating cell nuclear antigen (PCNA) immunolocalization in paraffin sections: An index of cell proliferation with evidence of deregulated expression in some, neoplasms. J Pathol.

[29] Knudson CM, Korsmeyer SJ (1997). Bcl-2 and Bax function independently to regulate cell death. Nat Genet.

[30] Subramanian A, Tamayo P, Mootha VK, Mukherjee S, Ebert BL, Gillette MA, Paulovich A, Pomeroy SL, Golub TR, Lander ES, Mesirov JP (2005). Gene set enrichment analysis: a knowledge-based approach for interpreting genome-wide expression profiles. Proc Natl Acad Sci USA.

