# Supplementary material for: Expression of HNF4G and its potential functions in lung cancer
Source: Oncotarget. 2017 Dec 4;9(26):18018–28. doi: 10.18632/oncotarget.22933 (PMC5915054; doi:10.18632/oncotarget.22933)
Supplement: Supplementary file 1 [file oncotarget-09-18018-s001.pdf]

Expression of HNF4G and its potential functions in lung cancer

SUPPLEMENTARY MATERIALS

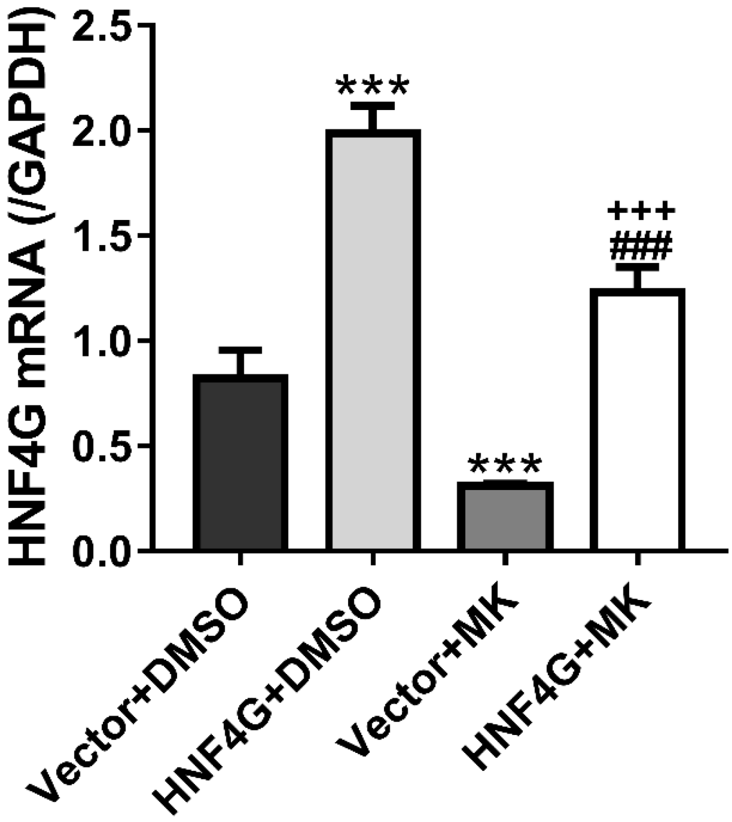

Supplementary Figure 1: A549 cells were infected with control virus (Vector) or HNF4G virus. HNF4G mRNA expression was determined in the present of either DMSO or AKT inhibitor (MK-2206, 2 µM). $^{***}P < 0.001$ versus Vector+DMSO; $^{###}P < 0.001$ versus HNF4G+DMSO; $^{+++}P < 0.001$ versus Vector+MK-2206.
